# Supplementary material for: Evaluation of a CZT-based photon-counting detector CT prototype for low-dose lung cancer screening using patient-specific lung phantoms
Source: Res Sq. 2026 Jul 9:rs.3.rs-9473696. Preprint. [Version 1] doi: 10.21203/rs.3.rs-9473696/v1 (PMC13370630; doi:10.21203/rs.3.rs-9473696/v1)
Supplement: 1 [file NIHPPRS9473696V1-supplement-1.pdf]

Supplemental Table 1 Scan settings for patient exams used for phantom preparation

	Part-solid nodule	Part-solid mass	Solid nodule	Solid mass	Ground-glass nodule small	Ground-glass nodule large
Tube Voltage [kVp]	100	120	100	120	100	120
Tube Current/ref * [mAs]	65/90	124/78	212/108	130/80	139/108	122 /80
CTDIvol [mGy]	2.68	6.69	6.73	7	4.44	6.55
Spiral pitch factor	1.5	2.6	2	1.55	2	1.95
Collimation [mm]	38.4	57.6	57.6	57.6	57.6	57.6
Kernel	Br51f	Br49d	Br49d	Br49d	Br49d	Br49d
Pixel spacing [mm]	0.57	0.77	0.77	0.71	0.77	0.85

Slice thickness [mm]	1.0	1.0	1.0	1.0	1.0	1.0
Field of view size [mm]	290	396	395	363	397	435
Matrix size	512	512	512	512	512	512

*Scans were tube current modulated.
